# A review of trauma and orthopaedic randomised clinical trials published in high-impact general medical journals

**DOI:** 10.1007/s00590-021-03137-3

**Published:** 2021-10-06

**Authors:** Luke Farrow, William T. Gardner, Andrew D. Ablett, Vladislav Kutuzov, Alan Johnstone

**Affiliations:** 1 Institute of Applied Health Sciences, University of Aberdeen, Foresterhill, Aberdeen, AB25 2ZD Scotland, UK; 2 Aberdeen Royal Infirmary, Aberdeen, UK; 3 Edinburgh Royal Infirmary, Edinburgh, UK; 4 Charing Cross Hospital, London, UK

**Keywords:** Trauma, Orthopaedics, Trial, RCT, Quality, Review

## Abstract

**Introduction:**

The recent past has seen a significant increase in the number of trauma and orthopaedic randomised clinical trials published in “the big five” general medical journals. The quality of this research has, however, not yet been established.

**Methods:**

We therefore set out to critically appraise the quality of available literature over a 10-year period (April 2010–April 2020) through a systematic search of these 5 high-impact general medical journals (JAMA, NEJM, BMJ, Lancet and Annals). A standardised data extraction proforma was utilised to gather information regarding: trial design, sample size calculation, results, study quality and pragmatism. Quality assessment was performed using the Cochrane Risk of Bias 2 tool and the modified Delphi list. Study pragmatism was assessed using the PRECIS-2 tool.

**Results:**

A total of 25 studies were eligible for inclusion. Over half of the included trials did not meet their sample size calculation for the primary outcome, with a similar proportion of these studies at risk of type II error for their non-significant results. There was a high degree of pragmatism according to PRECIS-2. Non-significant studies had greater pragmatism that those with statistically significant results (*p* < 0.001). Only 56% studies provided adequate justification for the minimum clinically important difference (MCID) in the population assessed. Overall, very few studies were deemed high quality/low risk of bias.

**Conclusions:**

These findings highlight that there are some important methodological concerns present within the current evidence base of RCTs published in high-impact medical journals. Potential strategies that may improve future trial design are highlighted.

**Level of evidence:**

Level 1.

**Supplementary Information:**

The online version contains supplementary material available at 10.1007/s00590-021-03137-3.

## Introduction

Evidence-based medicine is the established bedrock of good clinical care. Whilst historically there have been concerns over the strength of evidence base behind orthopaedic interventions [1], the recent past has seen an increase in the number of randomised clinical trials (RCT’s) published in major medical journals, particularly the so-called big five [2]. Understanding the strengths and limitations of these trials is vital to understanding their clinical applicability, as well as providing a key learning opportunity for future trial design and development.

Growth of the orthopaedic trial community has led to increasing interest in the concept of pragmatic trials, where the focus is to reflect real-world applicability of an intervention rather than providing causative explanations for trial outcomes. There have, however, been concerns raised about a risk of overgeneralisation, and associated scepticism about applicability to every circumstance [3, 4] associated with a pragmatic trial design.

The adequacy of reporting [5], design [6] and robustness [7] of clinical trauma and orthopaedic trials have also previously been called into question. Recent analysis [8] from trials published within a specific mainstream orthopaedic journal has identified general improvements in the quality and quality of analyses over time, but with trends towards smaller, single centre trial design.

However, several larger trials are now reported in high-impact non-orthopaedic medical journals, and thus were excluded from this previous analysis. As a result, there is little currently understood about the specifics of design, conduct and reporting related to these large-scale trauma and orthopaedic trials published in major general medical journals with a high impact factor.

We therefore set out to examine the quality of evidence produced from RCT’s published within this setting. Given the high-impact and international influence of these journals it is integral that the literature produced is of sound methodological quality with low risk of bias in order to provide substantial high-quality evidence for interventions.

## Materials and methods

### Study selection

A systematic search of 5 major high-impact general medical journals (colloquially known as “the big five”—British Medical Journal (BMJ), Journal of American Medical Association (JAMA), New England Journal of Medicine (NEJM), the Lancet and Annals of Internal Medicine (Annals)) was performed from April 2010–April 2020 using online bibliographical archives on each journal website. These journals have a combined mean impact factor of 46.8 (https://academic-accelerator.com/Impact-Factor-IF/), far in excess of any trauma and orthopaedic speciality journal. They have previously been used to examine adequacy of trial design in other areas of healthcare and provide a gold standard reference for trial quality, given their exclusivity and publication standards [9]. Screening of full text articles was performed by one author (VK) and verified by another (LF). All articles pertaining to any area of trauma and orthopaedics that described a surgical treatment-based intervention randomised clinical trial were included. Those articles describing other surgical fields, or pertaining specifically to non-surgical interventions, were excluded.

### Data collection

Data extraction was performed using a standardised proforma by three independent reviewers (VK, AA and TG). Any disagreement was mediated by a fourth individual (LF) until a communal decision was reached. Given the purpose of this study as a reflection of the available literature study authors were not contacted in the presence of missing data. All published or freely accessible data sources (for example, study protocols or trial monographs) for each study were, however, utilised. Data fields included in the analysis and extracted for each included trial are displayed in Table 1.

**Table 1:** Data fields

Trial design	Sample size calculation	Results
Article title	Significance level	Actual sample size (defined as the number of patients with primary outcome data at the follow-up time point specified in the trial methodology)
Year of publication	Power	Number of patients in intervention and control arms
Country of origin	Predicted effect size of minimum clinically important difference (MCID)	Outcomes in intervention and control arms
Funding source	Use of MCID	Significance level of result
Intervention group	Appropriate justification for predicted effect size/MCID (e.g. Pilot RCT, modelling study or Delphi process pertaining to that specific disease or injury)	Reporting of 95% confidence intervals
Control group	Reporting of the population standard deviation if MCID used	Number of patients lost to follow up (defined as the number of patients without primary outcome assessment at the follow-up time point specified in the trial methodology)
Study design (superiority vs non-inferiority)	Required sample size calculated for assessment of the primary outcome	Early termination
Pragmatic versus explanatory (Pragmatic Explanatory Continuum Indicator Summary—PRECIS 2 tool)		Unanticipated sample size modification within trial
Blinding		Cochrane risk of bias assessment
Anatomical Region		Delphi list
Trauma vs Elective		
Number of centres involved		
International study		
Primary outcome		
Type of primary outcome (mortality vs complication vs patient reported outcome or functional score)		

### Statistical analysis

Descriptive analyses of overall trial characteristics were performed. N (%) was calculated for categorical variables, with median values and range presented for continuous variables given these were all non-normally distributed.

Comparison for the predicted control group event rate (identified from sample size calculation) versus the actual control group event rate was made for dichotomous outcomes. Assessment of study pragmatism was compared between significant and non-significant results utilising an unpaired 2-tailed *t*-test.

Assessment regarding risk of bias was performed for each trial using two measures:

1. The Cochrane Risk of Bias 2 tool [10], with each study summarised as either low, medium or high risk of bias.
2. The modified Delphi list [11], with a maximum score of 9 points. Only items assessed with a “yes” were given a score of 1 point. For the purposes of the study, scores 8–9 were considered high quality, scores 5–7 medium quality, scores 4–6 low quality and scores 1–3 very low quality.

Characterisation of individual article post-publication data was also performed. This included the study Altmetric attention score where available (www.altmetric.com/about-our-data/the-donut-and-score) and number of citations (https://scholar.google.co.uk/).

All statistical analyses were performed using R (R: A language and environment for statistical computing. R Foundation for Statistical Computing, Vienna, Austria) and Microsoft Excel (Microsoft Corporation. (2018). Microsoft Excel, Washington, USA).

## Results

Full details of the extracted information are located in supplementary tables 1–3. The summary results are displayed in Table 2. Overall, we identified 25 studies suitable for inclusion [12–36]. Of these studies, 9 pertained to trauma, 3 to elective hip surgery, 7 to elective knee surgery, 4 to spinal surgery and 2 to elective shoulder surgery. A greater proportion of trials—16/25 (64%) were identified in the latter half of the study period (2016–2020), than the early period—9/25 (36%). Most studies were lead from the UK 12/25 (48%), with 9/12 (75%) of these funded by the National Institute of Health Research. Patient recruitment was performed from a median of 9 centres (range 1–81). Blinding was present in approximately half of trials 13/25 (52%); of these, 8/25 (32%) were single (assessor) blinded, and 5/25 (20%) were double blinded (patient and assessor). Regarding outcome assessment, the vast majority—22/25 (88%) utilised patient-reported outcome measures or functional scores as the primary outcome measure. Complication rate was only utilised in 3/25 (12%) trials as the primary outcome measure. No trials used mortality as the primary endpoint.

**Table 2:** Summary of results

Study characteristics									
Identified in latter half of study period (2016–2020) no. (%)	Superiority no. (%)	Pragmatic no. (%)	Any form of blinding no. (%)	Single assessor blinding no. (%)	Double assessor blinding no. (%)	Median no. centres (range)	International recruitment no. (%)	PROMs as primary outcome no. (%)	Complication rate as primary outcome no. (%)
16/25 (64)	23/25 (92)	18/25 (72)	13/25 (52)	8/25 (32)	5/25 (20)	9 (1–81)	2/25 (8)	22/25 (88)	3/25 (12)

Sample size									
Power data reported no. (%)	80% power value no. (%)	90% power value no. (%)	81.5% power value no. (%)	Used MCID for power calculation no. (%)	Justification for MCID or predicated effect size no. (%)	Reported SD required for sample size calculation no. (%)	Achieved target sample size for assessment of primary outcome no. (%)	Made adjustments to sample size calculation during trial no. (%)	
23/25 (92)	15/23 (65.2)	7/23 (30.4)	1/23 (4.4)	23/25 (92)	14/25 (56)	15/23 (65.2)	11/25 (44)	5/24 (20.8)	

Results						
Statistically significant (*p* < 0.05) no. (%)	Trials with a non-significant result and underpowered no. (%)	Trials with dichotomous outcomes allowing for assessment of FI/RFI no. (%)	HEALTH study RFI (LTFU)	FAITH study RFI (LTFU)	WHIST study RFI (LTFU)	Potential for overturning of results depending on results of LTFU no. (%)
7/25 (28)	10/18 (55.6)	3/25 (12)	10 (29)	8 (383)	7 (29)	3/3 (100)

Comparison for the predicted control group event rate versus the actual control group event rate		
HEALTH study	FAITH study	WHIST study
4.9% vs 8.3%	25% vs 21.8%	15% vs 6.7%

Cochrane risk of bias				
Low risk no. (%)	Some risk no. (%)	High risk no. (%)	High risk due to deviations from intended interventions no. (%)	Some risk of bias due to use of PROMs without patient blinding no. (%)
3/25 (12)	18/25 (72)	4/25 (6)	3/4 (75)	16/25 (64)

Regarding specific journals, 7 articles were published in the BMJ, 7 in the NEJM, 6 in the JAMA, 5 in the Lancet and none in the Annals. The mean Altmetric attention score© was 242 (range 22–681), and the mean number of citations per article was 230 (range 22–743).

### Study pragmatism

The PRECIS-2 (Pragmatic Explanatory Continuum Indicator Summary—2) tool[37] was utilised to assess the pragmatism of included studies. Scores for individual domains in each trial are displayed in supplementary table 4. Overall, there was a high degree of pragmatism identified (mean aggregated score across all studies and domains 4.2/5). Studies with statistically significant results had a lower mean overall PRECIS-2 score compared to those with non-significant results (mean 3.71 vs 4.4, respectively; *p* < 0.001).

### Sample size

All studies were set significance as *p* < 0.05. For studies with power data available, 15/23 (65.2%) utilised a power value of 80%, 7/23 (30.4%) a power value of 90%, and 1/23 (4.4%) a power value of 81.5%. Twenty-three out of 25 studies (92%) reported use of the MCID in order to perform their sample size calculation; however, only 14/25 studies (56%) had appropriate justification for use of the MCID or predicted effect size, and only 15/23 (65.2%) reported standard deviation in outcome for the target population when using MCID that is required to perform appropriate sample size calculations. Furthermore, only 14/25 studies (56%) achieved their target sample size for assessment of the primary outcome. Five out of 24 studies (20.8%) made amendments to the sample size calculation while the trial was ongoing.

### Results

Seven out of 25 (28%) trials reported statistically significant results (*p* < 0.05) for the primary outcome. Seven out of 18 (38.9%) of those trials that reported non-significant results had an actual sample size for the primary outcome smaller than the predicted sample size, indicating a potential type II error. Only 3 trials reported dichotomous outcomes that allowed for assessment of the FI/RFI and comparison of the predicted control group event rate versus the actual control group event rate. Given all three reported non-significant results, the RFI was utilised. For the HEALTH study [16], the RFI was 10, with loss to follow-up (LTFU) of 29 patients. For the FAITH study [29], the RFI was 8, with LTFU of 383 patients. For the WHIST study [21], the RFI was 7, with LTFU of 29 patients. Results of all three trials could have been overturned dependent on the results of those lost to follow-up. With regard to the comparison of the predicted control group event rate versus the actual control group event rate, both the WHIST and HEALTH studies had differences > 50% (Table 2).

### Risk of bias

The summary results for the Cochrane risk of bias analysis, including assessment for each included domain, are displayed in Table 3. Three out of 25 (12%) trials were adjudged to be at low risk of bias, 18/25 (72%) trials at some risk of bias, and 4/25 (6%) at high risk of bias. Three out of 4 (75%) of those studies judged at high risk of bias were due to deviations from the intended interventions. Sixteen out of 25 (64%) studies had at least some risk of bias in outcome measurement attributable to the use of PROMs without patient blinding where the outcome may have been influenced by knowledge of the intervention received. A summary bar chart of the percentage of risk by category is displayed in Supplementary Fig. 1.

**Table 3:**
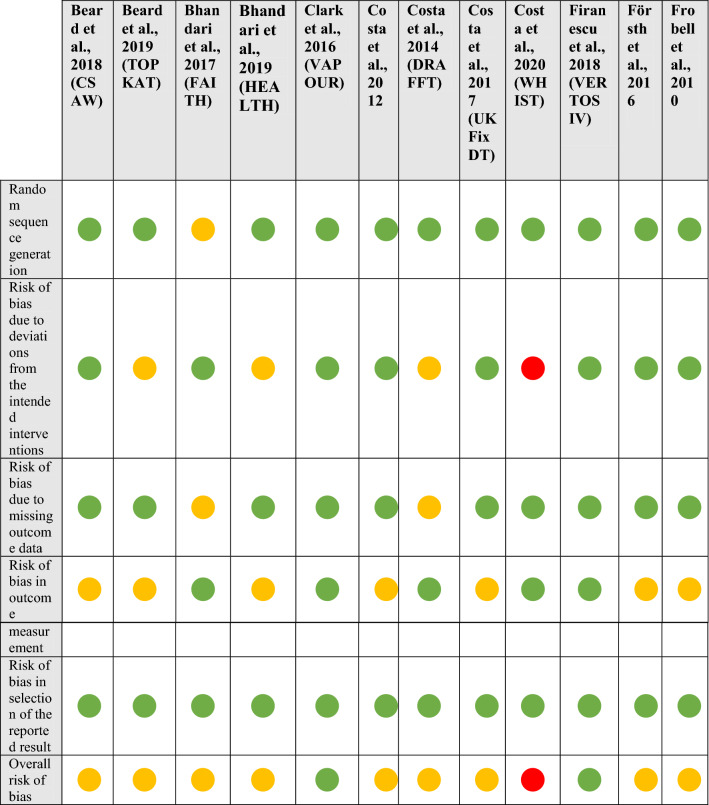

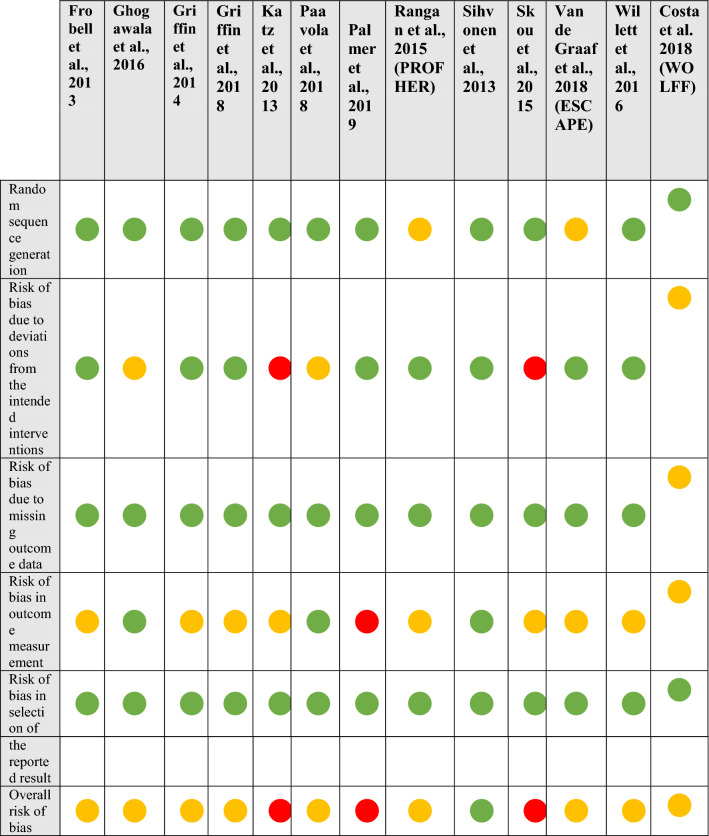
Revised Cochrane risk-of-bias tool for randomised trials (RoB 2)

Green circle = Low risk, Yellow circle = Some risk, Red circle = High risk

The summary results for the Delphi list assessment are displayed in Table 4. Five out of 25 (20%) trials were adjudged to be high quality, and 20/25 (80%) of trials were designated as medium quality. No trials were assessed to be of either low or very low quality. The main reason for designation of lower study quality was lack of blinding to patient, care provider or assessor.

**Table 4:** Delphi list assessment

	1. Treatment allocation a) Was a method of randomisation performed?	1. Treatment allocation b) Was treatment allocation concealed?	2. Were the groups similar at baseline regarding the most important prognostic factors?	3. Were the eligibility criteria specified?	4. Were the outcome assessors blinded?	5. Was the care provider blinded?	6. Were point estimates and measures of variability presented for the primary outcome measures?	7. Did the analysis include an intention-to-treat analysis?	Overall score /9
Frobell et al. [12]	Yes	No	Yes	Yes	No	No	Yes	Yes	6
Costa et al. [26]	Yes	No	Yes	Yes	Yes	No	Yes	Yes	7
Sihvonen et al. [13]	Yes	Yes	Yes	Yes	Yes	No	Yes	Yes	8
Frobell et al. [23]	Yes	No	Yes	Yes	No	No	Yes	Yes	6
Katz et al. [17]	Yes	No	Yes	Yes	No	No	Yes	Yes	6
Griffin et al. [23]	Yes	No	Yes	Yes	Yes	No	Yes	Yes	7
Costa et al. [35]	Yes	No	Yes	Yes	Yes	No	Yes	Yes	7
Rangan et al. [32]	Yes	No	Yes	Yes	Yes	No	Yes	Yes	7
Skou et al. [18]	Yes	No	Yes	Yes	Yes	No	Yes	Yes	7
Ghogawala et al. [14]	Yes	No	Yes	Yes	No	No	Yes	No	5
Försth et al. [15]	Yes	No	Yes	Yes	No	No	Yes	No	5
Willett et al. [34]	Yes	No	Yes	Yes	No	No	Yes	No	5
Clark et al. [20]	Yes	Yes	Yes	Yes	Yes	No	Yes	Yes	8
Costa et al. [31]	Yes	Yes	Yes	Yes	No	No	Yes	Yes	7
Bhandari et al. 2017	Yes	No	Yes	Yes	Yes	No	Yes	Yes	7
Paavola et al. [25]	Yes	Yes	Yes	Yes	Yes	No	Yes	Yes	8
Firanescu et al. [27]	Yes	Yes	Yes	Yes	Yes	No	Yes	Yes	8
Beard et al. [28]	Yes	Yes	Yes	Yes	Yes	No	Yes	Yes	8
Griffin et al. [30]	Yes	No	Yes	Yes	Yes	No	Yes	Yes	7
Van der Graaf et al. [33]	Yes	No	Yes	Yes	Yes	No	Yes	Yes	7
Bhandari et al. [16]	Yes	No	Yes	Yes	No	No	Yes	Yes	6
Beard et al. [19]	Yes	No	Yes	Yes	No	No	Yes	Yes	6
Palmer et al. [24]	Yes	No	Yes	Yes	Yes	No	Yes	Yes	7
Costa et al. [21]	Yes	No	Yes	Yes	Yes	No	Yes	Yes	7
Costa et al. [36]	Yes	No	Yes	Yes	Yes	No	Yes	Yes	7

## Discussion

Key findings from this analysis included that only a very small proportion of trauma and orthopaedic RCT’s published in high-impact general medical journals were judged to be of high quality/low risk of bias. Many published trials did not achieve their target sample size for assessment of the primary outcome, and several did not describe appropriate techniques to justify use of the MCID for the intended intervention. We identified a high degree of study pragmatism, with a lower likelihood of statistically significant results for more pragmatic designs.

Despite these concerns, these studies were highly cited in the literature, with evidence for widespread dissemination according to Altmetric attention scores©. Knowledge of deficiencies in the design and reporting of trauma and orthopaedic RCT’s can assist in the planning of future trials to improve scientific rigour and ensure widespread clinical applicability.

### Patient-reported outcome measures

The vast majority of studies utilised PROMs, with the MCID as the primary method of defining the delta within the sample size calculation. The MCID is an important concept; however, there are a few potential issues that need to be addressed when considering its use in this context. The first is that there is no universal definition of how best to evaluate the MCID. The MCID produced varies by technique used and depends on the patient’s baseline status, as well as study context [38]. Age is perhaps the most predominant example of this is, having previously been shown to influence baseline PROMs and response to surgical interventions for knee arthritis [39]. Use of PROMs with ceiling effects is also known to impart bias on outcomes following trauma and orthopaedic randomised clinical trials [40]. Given the identified widespread use of MCID, it is imperative future trials utilise appropriate techniques to ensure correct definition of the MCID in the population to be tested by the intervention.

It was notable that a significant proportion of trials (44%) did not achieve their target sample size for calculation of the primary outcome at the prespecified time point. This suggests that current estimates regarding participant retention are often incorrect. Overall, there was mean underestimation of eventual study sample size available for the primary outcome by approximately 5%, but this was as high as 28% in the studies by Frobell et al. [12] and Försth et al. [15], and above 10% in a number of others [14, 24, 25, 36]. Further careful consideration of factors potentially influential towards ongoing involvement or crossover is required during sample size calculation to ensure that sufficient recruitment. Guidelines for the conduct and reporting of RCT sample size calculations (DELTA 2 [41]) have previously been described and should be utilised to ensure a high probability of a study achieving its primary aim.

### Sample size

Identified smaller than predicted sample sizes are a concern for those trials with negative results (38.9%), where there is an associated risk of type II error. It is therefore difficult to determine whether the results for these trials were due to absence of evidence or actual evidence of absence of effect. We also identified significant differences in the predicted and actual effect size in the control group for included studies, which may additionally influence the ability to perform accurate outcome assessment. Future use of adaptive trial designs may help to eliminate some of these issues [42] and minimise research that does not achieve its desired intention [8].

### Study pragmatism

Another notable finding from our results was the high degree of pragmatism (according to PRECIS-2) identified in the included trials and the fact that a greater degree of pragmatism in approach was associated with lower likelihood of a significant result. Other research has previously highlighted how questions over the routine use of pragmatic trials may have had a role to play in the lack of translation from some trials towards change in clinical practice [43] and issues with trial recruitment [4, 44]. Surgeon and patient preference have both been shown to be influential in recruitment and retention to trauma and orthopaedic pragmatic RCTs [45]. It is vital that equipoise within the surgical community is established prior to embarking on any pragmatic trial, as recruitment bias and crossover remain a major concern. Use of the readiness assessment for pragmatic trials (RAPT) model may provide one method of determining suitability of an intervention for testing in a pragmatic trial [46]. We could not find any evidence of this tool having been used in trauma and orthopaedic research to determine the suitability of previously conducted pragmatic trials.

### Alternative approaches to trial design

Another option that requires further exploration regarding utility in the domain of clinical Trauma & Orthopaedic research is a Bayesian approach to trial design. This technique has been increasingly used in the wider trial community [47, 48] and may provide significant potential benefit in the heterogeneous populations seen across the breadth of Trauma and Orthopaedics [49].

### Strengths and limitations

Strengths of the study include the in-depth assessment of study quality and design for gold-standard benchmarks regarding the current state of orthopaedic research. Potential limitations to our study include that it may be possible that higher-quality evidence and lower risk of bias are seen in studies contained within other journals, but this is contradictory to what has been previously reported [8]. Post hoc power calculations were not conducted due to known methodological issues with this approach [50].

### Applicability

We provide a summary of the literature with note of areas for improvement, but it should be clear that these concerns are not applicable to all included studies, and the use of many of the methods discussed such as the use of PROMs, the MCID and pragmatic trials is supported when utilised appropriately.

## Conclusions

The majority of trauma and orthopaedic RCTs published in high-impact major medical journals have evidence of significant knowledge dissemination, but some notable concerns related to study quality.

We suggest the following changes may assist in future publication of low-risk trials: International co-operation in the development and funding of large-scale multi-centre randomised trials, appropriate calculation of the relevant MCID for the study hypothesis with use of widely validated PROMs, measures to improve trial retention, blinding of participants to intervention allocation when utilising PROMs, prior assessment of community equipoise (with potential increased use of explanatory trials where appropriate), and potential use of Bayesian approaches to trial design.

Caution should be used in the interpretation of highly pragmatic trials as these appear less likely to be associated with statistically significant results, although the exact nature of this relationship is unclear.

## Supplementary Information

Below is the link to the electronic supplementary material.

Supplementary file1 (TIF 87 KB)

Supplementary file2 (DOCX 28 KB)

Supplementary file3 (DOCX 17 KB)

Supplementary file4 (DOCX 17 KB)

Supplementary file5 (DOCX 15 KB)

## Data Availability

Data are available on request. No code was utilised.
