# Supplementary figures and images for: A review of trauma and orthopaedic randomised clinical trials published in high-impact general medical journals
Source: Eur J Orthop Surg Traumatol. 2021 Oct 6;32(8):1469–79. doi: 10.1007/s00590-021-03137-3 (PMC9587938; doi:10.1007/s00590-021-03137-3)

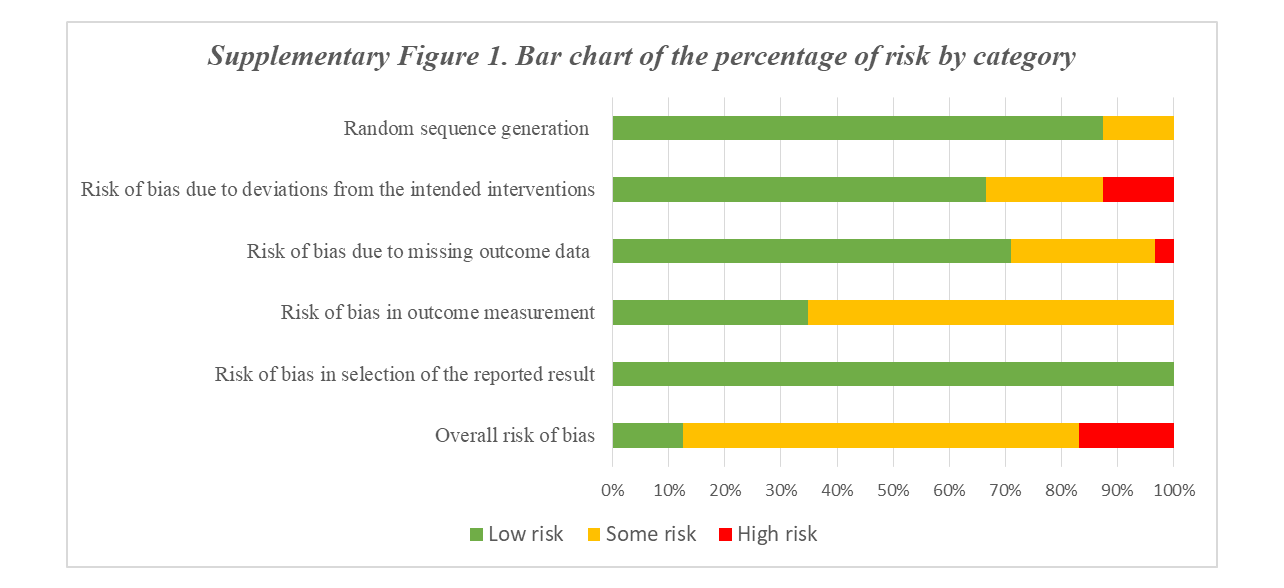

Supplement: Supplementary file 1 — Supplementary file1 (TIF 87 KB) [file 590_2021_3137_MOESM1_ESM.tif]
