# Supplementary material for: A review of trauma and orthopaedic randomised clinical trials published in high-impact general medical journals
Source: Eur J Orthop Surg Traumatol. 2021 Oct 6;32(8):1469–79. doi: 10.1007/s00590-021-03137-3 (PMC9587938; doi:10.1007/s00590-021-03137-3)
Supplement: Supplementary file 2 — Supplementary file2 (DOCX 28 KB) [file 590_2021_3137_MOESM2_ESM.docx]

| **Supplementary Table 1: Design** | | | | | | | | | | |  |
| --- | --- | --- | --- | --- | --- | --- | --- | --- | --- | --- | --- |
| **Short Title** | **Journal** | **Funding source** | **Country of origin** | **Intervention** | **Control** | **Trial Design** | | | **Anatomical Region e.g. Shoulder** | **Number of Centres involved** | **Altmetric© Score (citatations)** |
| **Frobell 2010** | NEJM | Multiple sources | Sweden | Structured Rehabilitation + Early ACL reconstruction | Structured Rehabilitation + optional delayed ACL reconstruction | Superiority | Non-Blind | Trauma | Knee | 2 | N/A (743) |
| **Costa 2012** | BMJ | NIHR | United Kingdom | Hip resurfacing arthroplasty | Total hip replacement | Superiority | Single Blind | Elective | Hip | 1 | 23 (64) |
| **Sihvonen 2013** | NEJM | Multiple sources | Finland | Arthroscopic Partial Meniscectomy | Sham Surgery | Superiority | Double blind | Elective | Knee | 5 | N/A (649) |
| **Frobell 2013** | BMJ | Multiple sources | Sweden | Early ACL reconstruction with structured rehabilitation | Delayed ACL repair with structured rehabilitation | Superiority | Non-Blind | Trauma | Knee | 2 | 376 (391) |
| **Katz 2013** | NEJM | NIH | United States | Arthroscopic Partial Meniscectomy + Physical Therapy | Physical Therapy alone | Superiority | Non-Blind | Elective | Knee | 7 | N/A (576) |
| **Griffin 2014** | BMJ | Arthritis Research UK | United Kingdom | ORIF of intra-articular calcaneal fractures | Non-operative treatment | Superiority | Single Blind | Trauma | Foot | 22 | 22 (231) |
| **Costa 2014** | BMJ | NIHR | United Kingdom | K-wire fixation | Plate fixation | Superiority | Single Blind | Trauma | Wrist | 18 | 42 (159) |
| **Rangan 2015** | JAMA | NIHR | United Kingdom | Surgery - replacement or fixation | Sling | Superiority | Non-Blind | Trauma | Humerus | 32 | 266 (286) |
| **Skou 2015** | NEJM | Multiple sources | Denmark | Total Knee Replacement + nonsurgical treatment | Nonsurgical treatment alone | Superiority | Non-Blind | Elective | Knee | 2 | N/A (394) |
| **Ghogawala 2016** | NEJM | Multiple sources | United States | Laminectomy + Fusion | Laminectomy Alone | Superiority | Non-Blind | Elective | Spine | 5 | N/A (426) |
| **Försth 2016** | NEJM | Multiple sources | Sweden | Decompression + Fusion (spondylolisthesis/no spondylolisthesis) | Decompression Alone (spondylolisthesis/no spondylolisthesis) | Superiority | Non-Blind | Elective | Spine | 7 | N/A (485) |
| **Willett 2016** | JAMA | NIHR | United Kingdom | Fixation | Cast immobilisation | Equivalence | Single Blind | Trauma | Ankle | 24 | 284 (74) |
| **Clark 2016** | Lancet | CareFusion Corporation | Australia | Vertebroplasty | Sham Surgery | Superiority | Double blind | Trauma | Spine | 4 | N/A (237) |
| **Costa 2017** | JAMA | NIHR | United Kingdom | Intramedullary nail | Locking plate | Superiority | Non-Blind | Trauma | Tibia | 28 | 28 (24) |
| **Bhandari 2017** | Lancet | Multiple sources | Canada | Sliding hip screw | Cancellous screw fixation | Superiority | Single Blind | Trauma | Hip | 81 ^i^ | N/A (22) |
| **Paavola 2018** | BMJ | Multiple sources | Finland | Arthroscopic subacromial decompression (ASD) | Diagnostic arthroscopy (DA) | Superiority | Double blind | Elective | Shoulder | 3 | 580 (77) |
| **Firanescu 2018** | BMJ | Stryker | Netherlands | Vertebroplasty | Sham procedure | Superiority | Double blind | Trauma | Spine | 4 | 394 (87) |
| **Beard 2018** | Lancet | Multiple sources | United Kingdom | Arthroscopic subacromial decompression (ASD) | Arthroscopy, (+ placebo) | Superiority | Double blind | Elective | shoulder | 30 | N/A (217) |
| **Griffin 2018** | Lancet | NIHR | United Kingdom | Arthroscopy | Personalised physiotherapy programme | Superiority | Single Blind | Elective | Hip | 23 | N/A (201) |
| **Van der Graaf 2018** | JAMA | Multiple sources | Netherlands | Arthroscopic Partial Meniscectomy | Physiotherapy | Non-Inferiority | Non-Blind | Elective | Knee | 9 | 681 (66) |
| **Bhandari 2019** | NEJM | Multiple sources | Canada | Total Hip Arthroplasty | Hemiarthroplasty | Superiority | Non-Blind | Trauma | Hip | 80 ^i^ | N/A (73) |
| **Beard 2019** | Lancet | NIHR | United Kingdom | Partial Knee Replacement | Total Knee Replacement | Superiority | Non-Blind | Elective | Knee | 27 | N/A (85) |
| **Palmer 2019** | BMJ | Multiple sources | United Kingdom | Arthroscopic hip surgery | Physiotherapy and activity modification | Superiority | Single Blind | Elective | Hip | 7 | 196 (99) |
| **Costa 2020** | JAMA | NIHR | United Kingdom | Incisional negative pressure wound therapy | Standard wound therapy | Superiority | Single Blind | Trauma | Lower limb | 24 | 146 (64) |
| **Costa 2018** | JAMA | NIHR | United Kingdom | Negative pressure wound therapy | Standard wound therapy | Superiority | Single Blind | Trauma | Lower limb | 24 | 104 (19) |
|  |  | | | | | | | | | |  |

N/A = Not available. NEJM = New England Journal of Medicine, JAMA = Journal of the American Medical Association, BMJ = British Medical Journal, NIHR = National Institute for Health Research, NIH = National Institutes of Health
