# Supplementary material for: A review of trauma and orthopaedic randomised clinical trials published in high-impact general medical journals
Source: Eur J Orthop Surg Traumatol. 2021 Oct 6;32(8):1469–79. doi: 10.1007/s00590-021-03137-3 (PMC9587938; doi:10.1007/s00590-021-03137-3)
Supplement: Supplementary file 3 — Supplementary file3 (DOCX 17 KB) [file 590_2021_3137_MOESM3_ESM.docx]

| **Supplementary Table 2: Outcomes** | | | | | | | | | |
| --- | --- | --- | --- | --- | --- | --- | --- | --- | --- |
| **Short Title** | **primary outcome** | **type of primary outcome - mortality (1), complication rate (2), functional score/PROM (3)** | **significance level** | **Power (%)** | **predicted effect size (delta)** | **Used minimal clinically important difference (MCID)? No (0), Yes (1)** | **IF MCID used: is predicted standard deviation of population reported? No (0), Yes (1), blank = NA** | **Justification for predicted delta? E.g. pilot study. No (0), Yes (1)** | **sample size required for primary outcome analysis** |
| **Frobell 2010** | Knee injury and Osteoarthritis Outcome Score (KOOS) at 2 years | 3 | not recorded | 80 | 10 point difference in KOOS at 2 years | 1 | 0 | 0 (based on an OA study) | 168 |
| **Sihvonen 2013** | Lysholm + Western Ontario Meniscal Evaluation Tool (WOMET) scores + Visual analogue scale (VAS) pain scores at 12 months | 3 | 0.05 | 80 | 11.5 point difference in Lysholm Score; 15.5 point difference in WOMET Score. 2.0 point difference in Pain Score | 1 | 1 | 1 (based on earlier prospective study) | 108 |
| **Ghogawala 2016** | 36-point Short Form Survey (SF-36) at 2 years | 3 | 0.05 | 80 | 7.5 point difference in SF-36 physical component summary scores | 1 | 0 | 1 (based on an earlier study of lumbar spine patients) | 64 |
| **Försth 2016** | Oswestry Disability index (ODI) at 2 years | 3 | 0.05 | 80 | 12 point difference in ODI score | 1 | 0 | 0 | 320 |
| **Bhandari 2019** | Secondary Hip Procedure within 24 months | 2 | 0.05 | 80 | "45% lower relative risk of the primary end point at 2 years in the hemiarthroplasty group than in the total hip arthroplasty group" | 0 | NA | 0 (based on survey of participating surgeons) | 1434 |
| **Katz 2013** | Western Ontario and McMaster Universities Osteoarthritis Index (WOMAC) score at 6 months | 3 | 0.05 | 80 | 10-point difference in WOMAC Score | 1 | 0 | 1 | 340 |
| **Skou 2015** | Four Knee Injury and Osteoarthritis Outcome Score subscales (KOOS4) at 12 months | 3 | 0.05 | 90 | 10-point difference in KOOS4 at 12 months | 1 | 0 | 0 (based on what is commonly used) | 82 |
| **Beard 2019** | Oxford Knee Score at 5 years | 3 | 0.05 | 80 | 2.0-point difference in Oxford Knee Scores | 1 | 1 | 1 | 500 |
| **Clark 2016** | Pain score >4/10 at 14 days | 3 | 0.05 | 80 | Unclear | 1 | 0 | 0 | 120 |
| **Costa 2020** | Deep surgical site infection at 30 days diagnosed according to the criteria from the US Centers for Disease Control and Prevention | 2 | 0.05 | 90% | 6% reduction of deep surgical site infection | 1 | 0 | 0 (based on survey data from UK T&O society, 120 surgeons) | 1230 |
| **Frobell 2013** | Four Knee Injury and Osteoarthritis Outcome Score subscales (KOOS4) at 5 years | 3 | 0.05 | 80% | 10 point difference in KOOS4 | 1 | 1 | 0 (used predicted effect size based on different PROM calculation) | 120 |
| **Griffin 2014** | Kerr-Atkins score at 2 yrs | 3 | 0.05 | 80% | 15 points difference in Kerr-Atkins Score | 1 | 1 | 1 (based on discussion with collaborators from a pilot study of 24 patients ) | 126 |
| **Palmer 2019** | Hip outcome score activities of daily living subscale (HOS ADL) at 8 months | 3 | 0.05 | 90% | 9 point difference in HOS ADL Score | 1 | 1 | 1 (based on the literature) | 214 |
| **Paavola 2018** | Visual analogue scale (VAS) pain scores at 2 years | 3 | 0.05 | 90% | 15 points difference in VAS Scores | 1 | 1 | 1 (based on the literature) | 136 |
| **Costa 2012** | Oxford hip score and Harris hip score in 12 months | 3 | 0.05 | 80% | 5 point difference in OHS; 7 point difference in HHS | 1 | 1 | 1 (based on the literature) | 110 |
| **Firanescu 2018** | Visual analogue scale (VAS) pain scores at 12 months | 3 | 0.05 | 80% | 1.5 point difference in VAS Scores | 1 | 0 | 0 | 142 |
| **Beard 2018** | Oxford Shoulder Score (OSS) at 6 months | 3 | 0.05 | 90% | 4.5 difference in Oxford Shoulder Score | 1 | 1 | 1 (previous studies) | 170 |
| **Bhandari 2017** | Re-operation within 24 months | 2 | 0.05 | 81.5% | 25% Relative Risk Reduction | 0 |  | 1 (previous studies + pilot) | 1000 |
| **Griffin 2018** | International Hip Outcome Tool (IHOT) score at 12 months | 3 | 0.05 | 90% | 6.1 point difference in IHOT score | 1 | 1 | 1 (pilot) | 344 |
| **Costa 2017** | Disability Rating Index (DRI) at 6 months | 3 | 0.05 | 90% | 8 point difference in DRI | 1 | 1 | 1 (pilot) | 264 |
| **Rangan 2015** | Oxford Shoulder Score (OSS) averaged over 24 months (6,12,24 months) | 3 | 0.05 | 80% | 5 point difference in OSS | 1 | 1 | 1 (observational data - authors own) | 200 |
| **Van der Graaf 2018** | International Knee Documentation Committee (IKDC) Score at 24months | 3 | 0.05 | 90% | 8 point difference in IKDC | 1 | 1 | 0 | 320 |
| **Willett 2016** | Olerud-Molander Ankle Score (OMAS) at 6 months | 3 | 0.05 | 80% | 6 point equivalence margin | 1 | 1 | 1 (single pilot phase) | 620 |
| **Costa 2014** | Patient-Rated Wrist Evaluation (PRWE) Score at 12 months | 3 | 0.05 | 80% | 6 point difference in PRWE Score | 1 | 1 | 0 | 350 |
| **Costa 2018** | Disability Rating Index (DRI) at 12 months | 3 | 0.05 | 90% | 8 point difference in DRI | 1 | 1 | 0 | 460 |
