# Supplementary material for: A review of trauma and orthopaedic randomised clinical trials published in high-impact general medical journals
Source: Eur J Orthop Surg Traumatol. 2021 Oct 6;32(8):1469–79. doi: 10.1007/s00590-021-03137-3 (PMC9587938; doi:10.1007/s00590-021-03137-3)
Supplement: Supplementary file 4 — Supplementary file4 (DOCX 17 KB) [file 590_2021_3137_MOESM4_ESM.docx]

| **Supplementary Table 3: Results** | | | | | | | | | | | | | |
| --- | --- | --- | --- | --- | --- | --- | --- | --- | --- | --- | --- | --- | --- |
| **Short Title** | **actual sample size** | **Sample size actual < required No (0), Yes (1)** | **number of patients in intervention arm** | **number of patients in control arm** | **number events / score of primary outcome intervention arm** | **number events / score of primary outcome control arm** | **actual effect size (delta)** | **standard deviation or 95% CI for delta** | **significance level of result** | **sig <0.05 1 = Yes, 0=No** | **number of patients lost to follow up** | **study terminated early? No (0), 1 (Yes)** | **sample size modification within trial? No (0), 1 (Yes)** |
| **Frobell 2010** | 121 | 1 | 62 | 59 | 39.2 (34.5 to 43.8) | 39.4 (34.6-44.1) | 0.18 | −6.5 to 6.8 | 0.96 | 0 | 20 | 0 | 1 |
| **Sihvonen 2013** | 146 | 0 | 70 | 76 | Lysholm 21.7 (17.6-25.8), WOMET 24.6 (19.7-29.4), Pain 3.1 (2.5-3.8) | Lysholm 23.3 (19.5-27.2), WOMET 27.1 (22.4-31.8), Pain 3.3 (2.8-3.8) | Lysholm -1.6, WOMET -2.5, Pain -0.1 | Lysholm: −7.2 to 4.0; WOMET: −9.2 to 4.1; Pain −0.9 to 0.7 | not reported | 0 | 0 | 0 | 0 |
| **Ghogawala 2016** | 57 | 1 | 28 | 29 | 15.2 (10.9-19.5) | 9.5 (5.2-13.8) | 5.7 | 0.1 to 11.3 | 0.046 | 1 | 9 | 0 | 0 |
| **Försth 2016** | 228 | 1 | 111 | 117 | 27 | 24 | 3 | SD 2.45 | 0.24 | 0 | 19 | 0 | 1 |
| **Bhandari 2019** | 1441 | 0 | 718 | 723 | 57 | 60 | 3; HR 0.95 | 0.64 to 1.40 | 0.79 | 0 | 383 | 0 | 0 |
| **Katz 2013** | 330 | 1 | 161 | 169 | 20.9 (17.9-23.9) | 18.5 (15.6-21.5) | 2.4 | -1.8 to 6.5 | 0.2645 | 0 | 21 | 0 | 0 |
| **Skou 2015** | 100 | 0 | 50 | 50 | 32.5 (26.6-38.3) | 16 (10.1-21.9) | 16.5 | 10.2 to 22.7 | 0.0001 | 1 | 0 | 0 | 0 |
| **Beard 2019** | 464 | 1 | 233 | 231 | 38 (SD 10.1) | 37 (SD 10.6) | 1.04 | -0.42 to 2.50 | 0.159 | 0 | 64 | 0 | 0 |
| **Clark 2016** | 120 | 1 | 55 | 57 | 24 (44%) | 12 (21%) | 23 | 6 to 39 | 0.011 | 1 | 8 | 0 | 0 |
| **Costa 2020** | 1519 | 0 | 770 | 749 | 45 | 50/749 | −0.77% | −3.19% to 1.66% | 0.52 | 0 | 29 | 0 | 0 |
| **Frobell 2013** | 120 | 1 | 61 | 59 | 42.9 | 44.9 | -2 | −8.5 to 4.5 | 0.54 | 0 | 1 | 0 | 0 |
| **Griffin 2014** | 143 | 0 | 69 | 74 | 69.8 (raw score) | 65.7 | -0.03 points | −7.08 to 7.02 | 0.993 | 0 | 0 | 0 | 0 |
| **Palmer 2019** | 188 | 1 | 100 | 88 | 78.4 (raw score) | 69.2 | 10 points | 6.4 to 13.6 | 0.001 | 1 | 34 | 0 | 1 |
| **Paavola 2018** | 118 | 1 | 59 | 59 | Rest = 36, activity = 55.4 | Rest = 31.4; activity = 47.5 | rest: −4.6 points; activity = −9.0 points | rest: −11.3 to 2.1; activity: −18.1 to 0.2 | 0.054 | 0 | 4 | 0 | 0 |
| **Costa 2012** | 126 | 0 | 60 | 66 | OHS = 40.4; HHS = 88.4 (raw score) | OHS = 38.2; HHS = 82.3 | OHS = 2.23 points; HHS = 6.04 points | OHS: −1.52 to 5.98; HHS: −0.51 to 12.58 | 0.242 | 0 | 6 | 0 | 0 |
| **Firanescu 2018** | 176 | 0 | 90 | 86 | 5 | 4.75 | 0.13 points | –0.41 to 0.66 | not reported |  | 4 | 0 | 0 |
| **Beard 2018** | 209 | 0 | 106 | 103 | 32.7 | 34.2 | -1.3 | -3.9 to 1.3 | 0.3141 | 0 | 25 | 0 | 0 |
| **Bhandari 2017** | 1079 | 0 | 557 | 551 | 107 | 117 | 2.1 | 0.63 to 1.09 | 0.18 | 0 | 29 | 0 | 1 |
| **Griffin 2018** | 348 | 0 | 171 | 177 | 58.8 (SD 27) | 49.7 (SD 25) | 6.8 | 1.7 to 12.0 | 0.0093 | 1 | 27 | 0 | 0 |
| **Costa 2017** | 321 | 0 | 161 | 160 | 29.8 (26.0-33.7) | 33.8 (29.7-37.9) | 4 | -1 to 9 | 0.11 | 0 | 39 | 0 | 0 |
| **Rangan 2015** | 250 | 0 | 125 | 125 | 39.07 (37.30 to 40.76) | 38.32 (36.57 to 39.99) | 0.75 | −1.33 to 2.84 | 0.48 | 0 | 35 | 0 | 0 |
| **Van der Graaf 2018** | 321 | 0 | 159 | 162 | 26.2 (23.2 to 29.3) | 20.4 (17.5 to 23.2) | 5.8 | not reported | 0.04 | 1 | 32 | 0 | 1 |
| **Willett 2016** | 620 | 1 | 309 | 311 | 66 (63.6 to 68.5) | 64.5 (61.8 to 67.2) | −0.6 (−3.9 to 2.6) | -3.9 to 2.6 | 0.001* | 1 | 62 | 0 | 0 |
| **Costa 2014** | 415 | 0 | 211 | 204 | 15.3 (15.8 SD) | 13.9 (17.1 SD) | -1.3 | -4.5 to 1.8 | 0.398 | 0 | 29 | 0 | 0 |
| **Costa 2018** | 374 | 1 | 179 | 195 | 45.5 (28 SD) | 42.4 (24.2 SD) | -3.9 | -8.9 to 1.2 | 0.13 | 0 | 251 | 0 | 0 |

Willett et al provided p values for equivalence with regards to the primary outcome.
