# Supplementary material for: A review of trauma and orthopaedic randomised clinical trials published in high-impact general medical journals
Source: Eur J Orthop Surg Traumatol. 2021 Oct 6;32(8):1469–79. doi: 10.1007/s00590-021-03137-3 (PMC9587938; doi:10.1007/s00590-021-03137-3)
Supplement: Supplementary file 5 — Supplementary file5 (DOCX 15 KB) [file 590_2021_3137_MOESM5_ESM.docx]

**Supplementary Table 4 - PRECIS-2 assessment results**

| **Trial** | **Eligibility criteria** | **Recruitment path** | **Setting** | **Organisation intervention** | **Flexibility of experimental intervention – delivery** | **Flexibility of experimental intervention – adherence** | **Follow-up** | **Outcome** | **Analysis** | **Mean** |
| --- | --- | --- | --- | --- | --- | --- | --- | --- | --- | --- |
| **Frobell 2010** | 2 | 5 | 2 | 5 | 5 | - | 4 | 5 | 5 | 4.1 |
| **Costa 2012** | 5 | 5 | 1 | 5 | 5 | - | 4 | 5 | 5 | 4.4 |
| **Sihvonen 2013** | 3 | 5 | 5 | 5 | 1 | - | 4 | 5 | 5 | 4.1 |
| **Frobell 2013** | 2 | 5 | 2 | 5 | 5 | 5 | 3 | 5 | 5 | 4.1 |
| **Katz 2013** | 4 | 5 | 5 | 4 | 3 | 5 | 1 | 5 | 5 | 4.1 |
| **Griffin 2014** | 4 | 5 | 5 | 5 | 2 | - | 4 | 5 | 5 | 4.4 |
| **Costa 2014** | 4 | 5 | 5 | 5 | 5 | - | 4 | 5 | 5 | 4.8 |
| **Rangan 2015** | 2 | 5 | 5 | 5 | 5 | - | 3 | 5 | 5 | 4.4 |
| **Sjou 2015** | 3 | 5 | 1 | 3 | 2 | 1 | 1 | 5 | 5 | 2.9 |
| **Ghogawala 2016** | 2 | 5 | 5 | 5 | 1 | - | 4 | 5 | 1 | 3.5 |
| **Försth 2016** | 2 | 5 | 5 | 4 | 5 | - | 4 | 5 | 1 | 3.9 |
| **Willett 2016** | 4 | 5 | 5 | 4 | 5 | 5 | 5 | 5 | 3 | 4.6 |
| **Clark 2016** | 2 | 5 | 3 | 5 | 1 | - | 5 | 5 | 5 | 3.9 |
| **Costa 2017** | 5 | 5 | 5 | 5 | 5 | - | 5 | 5 | 5 | 5 |
| **Bhandari 2017** | 5 | 5 | 5 | 4 | 4 | - | 1 | 5 | 5 | 4.25 |
| **Paavola 2018** | 2 | 5 | 5 | 5 | 4 | - | 4 | 5 | 5 | 4.4 |
| **Firanescu 2018** | 5 | 4 | 5 | 5 | 1 | - | 2 | 5 | 5 | 4 |
| **Beard 2018** | 2 | 5 | 5 | 5 | 4 | - | 5 | 5 | 5 | 4.5 |
| **Griffin 2018** | 5 | 4 | 5 | 1 | 2 | 3 | 5 | 5 | 5 | 3.9 |
| **Van der Graaf 2018** | 2 | 5 | 5 | 4 | 2 | 5 | 4 | 5 | 5 | 4.1 |
| **Bhandari 2019** | 2 | 5 | 5 | 4 | 4 | - | 2 | 4 | 5 | 3.9 |
| **Beard 2019** | 4 | 5 | 5 | 4 | 5 | - | 3 | 5 | 5 | 4.5 |
| **Palmer 2019** | 4 | 5 | 5 | 1 | 3 | - | 4 | 5 | 5 | 4 |
| **Costa 2020** | 4 | 5 | 5 | 5 | 5 | 5 | 5 | 5 | 4 | 4.9 |
| **Costa 2018** | 4 | 5 | 5 | 5 | 5 | 5 | 4 | 5 | 5 | 4.8 |
